# Australian private practice metropolitan telepsychiatry during the COVID-19 pandemic: analysis of Quarter-2, 2020 usage of new MBS-telehealth item psychiatrist services

**DOI:** 10.1177/1039856220975294

**Published:** 2020-12-06

**Authors:** Jeffrey CL Looi, Stephen Allison, Tarun Bastiampillai, William Pring, Rebecca Reay

**Affiliations:** Academic Unit of Psychiatry and Addiction Medicine, Australian National University Medical School, Canberra Hospital, ACT, Australia; Private Psychiatry, ACT, Australia; College of Medicine and Public Health, Flinders University, SA, Australia; College of Medicine and Public Health, Flinders University, SA, Australia; Department of Psychiatry, Monash University, VIC, Australia; Monash University, and Centre for Mental Health Education and Research at Delmont Private Hospital, VIC, Australia; Private Psychiatry, VIC, Australia; Academic Unit of Psychiatry and Addiction Medicine, Australian National University Medical School, Canberra Hospital, ACT, Australia; Private Practice, ACT, Australia

**Keywords:** COVID-19, telepsychiatry, telehealth, psychiatrist, private practice

## Abstract

**Objective:**

The Australian Commonwealth Government introduced new psychiatrist Medicare-Benefits-Schedule (MBS)-telehealth items in the first wave of the COVID-19 pandemic to assist with previously office-based psychiatric practice. We investigate private psychiatrists’ uptake of (1) video- and telephone-telehealth consultations for Quarter-2 (April–June) of 2020 and (2) total telehealth and face-to-face consultations in Quarter-2, 2020 in comparison to Quarter-2, 2019 for Australia.

**Methods:**

MBS item service data were extracted for COVID-19-psychiatrist-video- and telephone-telehealth item numbers and compared with a baseline of the Quarter-2, 2019 (April–June 2019) of face-to-face consultations for the whole of Australia.

**Results:**

Combined telehealth and face-to-face psychiatry consultations rose during the first wave of the pandemic in Quarter-2, 2020 by 14% compared to Quarter-2, 2019 and telehealth was approximately half of this total. Face-to-face consultations in 2020 comprised only 56% of the comparative Quarter-2, 2019 consultations. Most telehealth provision was by telephone for short consultations of ⩽15–30 min. Video consultations comprised 38% of the total telehealth provision (for new patient assessments and longer consultations).

**Conclusions:**

There has been a flexible, rapid response to patient demand by private psychiatrists using the new COVID-19-MBS-telehealth items for Quarter-2, 2020, and in the context of decreased face-to-face consultations, ongoing telehealth is essential.

The Australian Commonwealth Government introduced Medicare-Benefits-Schedule (MBS) item numbers for video and telephone consultation in both metropolitan/non-regional areas for medical specialists, including psychiatrists, in response to the first wave of the COVID-19 pandemic in Australia.^1^ There has been rapid adoption by patients and psychiatrists of telehealth.^2^ Private practice psychiatry in Australia remains largely office based, and is estimated to provide 50%–60% of specialist psychiatric care.^3^ Accordingly, it is necessary to understand the impact of the COVID-19 pandemic on the overall demand for private practice consultation, and the uptake of the new telehealth MBS items as a key health policy for safe access to psychiatric care.

Part of a series of papers analysing Australia state data,^4,5^ this paper analyses the whole-of-Australia data for Quarter-2, 2020. We determine the amount of telehealth, as well as face-to-face office-based consultation during the 2020 introduction of these MBS items, compared to a baseline of Quarter-2, 2019 face-to-face consultation prior to COVID-19.

## Methods

MBS item service data were extracted from the Services Australia Medicare Item Reports (http://medicarestatistics.humanservices.gov.au/statistics/mbs_item.jsp) for practice office-based face-to-face (in-person) consultations (289, 291, 293, 296, 300, 302, 304, 306, 308, 342, 344, 346, 348, 350, 352). All MBS item descriptors were derived from http://www.mbsonline.gov.au/internet/mbsonline/publishing.nsf/Content/Home. Service data were extracted for psychiatrist video (92434, 92435, 92436, 92437, 91827, 91828, 91829, 91830, 91831, 92455, 92456, 92457, 92458, 92459, 92460) and telephone (92474, 92475, 92476, 92477, 91837, 91838, 91839, 91840, 91841, 92495, 92496, 92497, 92498, 92499, 92500) telehealth item numbers corresponding to the pre-existing in-person consultations.

Psychiatrist MBS item service data for Quarter-2 (April–June) 2020 in Microsoft Excel format were downloaded from Services Australia and transferred to a purpose-built Excel database and analysed using Excel (Microsoft Office Home and Student 2019; Microsoft Corporation, Seattle, Washington, USA). We extracted as a baseline comparator face-to-face data from Quarter-2 (April–June), 2019. Totals were calculated for combined video- and telephone-telehealth, as well as the combined sum of video- and telephone-telehealth and face-to-face consultations for Quarter-2, 2020. Video-telehealth consultations were calculated as a percentage of total of video- and telephone-telehealth consultations for Quarter-2, 2020. We calculated the percentages for combined (video and telephone) telehealth as a proportion of Quarter-2, 2019 face-to-face consultations and the sum total of video- and telephone-telehealth and in-person consultations for Quarter-2, 2020. Finally, the sum total of video- and telephone-telehealth and face-to-face consultations for Quarter-2, 2020 was calculated as a percentage of Quarter-2, 2019 face-to-face consultations.

## Results

Results are summarised below.

See Table 1 for overall data summary.

**Table 1.** Overall data summary

Face-to-face	F2F 2020	Video item	Video Tele2020	Telephone item	Tele Tele2020	F2F 2019	F2F20/19%	Vid + Tel2020	Vid + Tel + F2F2020	Vid/Total Teleh 2020%	TotalTelheal + F2F2020/F2F2019%	Tele health2020/Total Tele health F2F2020%	Total Tele health 2020/ F2F2019%	Tele health 2020/Total Telehealth + F2F2020%
289	38	92434	11	92474	6	74	51.35	17	55	64.71	74.32	30.91	22.97	30.91
291	6583	92435	1316	92475	1584	10,839	60.73	2900	9483	45.38	87.49	30.58	26.76	30.58
293	1375	92436	263	92476	644	2272	60.52	907	2282	29.00	100.44	39.75	39.92	39.75
296	21,606	92437	4625	92477	1752	29,958	72.12	6377	27,983	72.53	93.41	22.79	21.29	22.79
300	2948	91827	1491	91837	10,187	6322	46.63	11,678	14,626	12.77	231.35	79.84	184.72	79.84
302	25,886	91828	9831	91838	36,369	53,053	48.79	46,200	72,086	21.28	135.88	64.09	87.08	64.09
304	82,341	91829	29,205	91839	66,338	150,968	54.54	95,543	177,884	30.57	117.83	53.71	63.29	53.71
306	78,749	91830	47,005	91840	37,405	151,165	52.09	84,410	163,159	55.69	107.93	51.73	55.84	51.73
308	5128	91831	1936	91841	1746	8534	60.09	3682	8810	52.58	103.23	41.79	43.15	41.79
342	5501	92455	276	92495	153	7685	71.58	429	5930	64.34	77.16	7.23	5.58	7.23
344	24	92456	33	92496	0	66	36.36	33	57	100.00	86.36	57.89	50.00	57.89
346	453	92457	255	92497	29	1039	43.60	284	737	89.79	70.93	38.53	27.33	38.53
348	5687	92458	632	92498	919	7072	80.42	1551	7238	40.75	102.35	21.43	21.93	21.43
350	4184	92459	478	92499	452	5045	82.93	930	5114	51.40	101.37	18.19	18.43	18.19
352	7302	92460	1117	92500	2286	10,464	69.78	3403	10,705	32.82	102.30	31.79	32.52	31.79
**Total**	**247,805**		**98,474**		**159,870**	**444,556**	**55.74**	**258,344**	**506,149**	**38.12**	**113.85**	**51.04**	**58.11**	**51.04**

*Note.* Face-to-Face: Psychiatrist Office-Based Face-to-Face MBS-Item-Number: New patient assessment items are telehealth items corresponding to face-to-face consultations 289 (assessment of new patient with autism), 291 (comprehensive assessment and 12-month treatment plan), 293 (review of 291 plan), 296 (new patient for a psychiatrist or patient not seen in last two calendar years); Standard office-based consultation items are time-based items corresponding to face-to-face consultations: 300 (<15 minutes), 302 (15-30 minutes), 304 (30-45 minutes), 306 (45-75 minutes) and 308 (75 minutes plus; Group psychotherapy items equivalents: 342 (group psychotherapy 1 hour plus of 2-9 unrelated patients), 344 (group psychotherapy 1 hour plus of family of 3 patients) and 346 (group psychotherapy 1 hour plus of family group of 2 patients); Interview of a person other than the patient, for the care of the patient: 348 (initial diagnostic evaluation, 20-45 minutes), 350 (initial diagnostic evaluation, 45 minutes plus), and 352 (20 minutes plus, not exceeding 4 consultations).

**Figure 1.**
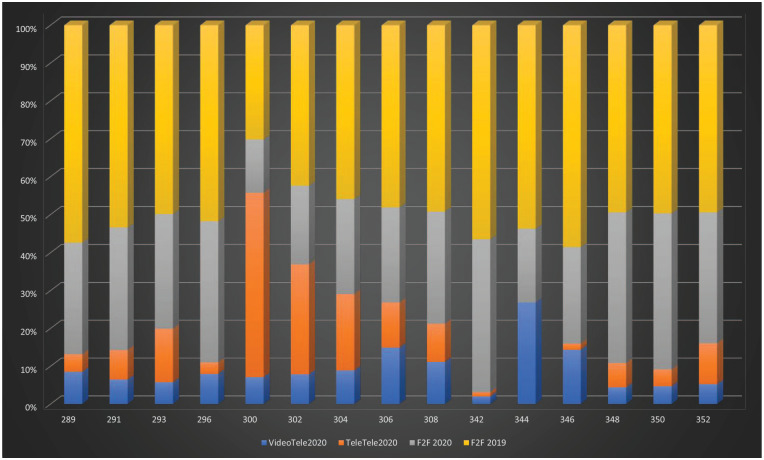
Quarter-2, 2020 individual psychiatrist MBS item usage by modality and year. VideoTele2020: video-telehealth count; TeleTele2020: tele-telehealth count; F2F2020: face-to-face consultations for Quarter-2, 2020: (count); F2F 2019: face-to-face consultations for Quarter-2, 2019 (count). X-axis: MBS-Item-Numbers (F2F, Video- and telephone-telehealth equivalents); Y-axis: percentage of total services.

See Figure 1 for Quarter-2 individual psychiatrist MBS item usage by modality and year.

**Figure 2.**
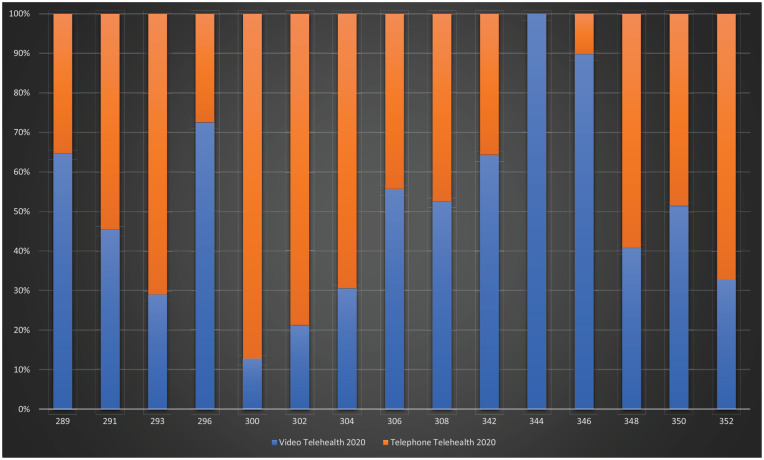
Quarter-2, 2020 video- vs telephone-telehealth. X-axis: MBS-Item-Numbers (Video- and telephone-telehealth equivalents); Y-axis: percentage of total telehealth consultations.

See Figure 2 for Quarter-2, 2020 video- vs telephone-telehealth.

## Discussion

For Quarter-2, 2020, the total combined use of telehealth and face-to-face consultations increased by 14% above the total of pre-COVID-19 Quarter-2, 2019 face-to-face consultations, reflecting a substantial increased demand for and provision of overall private psychiatry services (Table 1 and Figure 1).

This occurred in the context of a reduction in face-to-face consultation during Quarter-2, 2020, representing 56% of the face-to-face consultations for Quarter-2, 2019. Face-to-face consultations were most used for new patient assessment (items: 291, 293, 296) and longer consultations ⩾30 min (items: 304, 306, 308).

Overall, video- and telephone-telehealth constituted 51% of the combined total of telehealth and face-to-face consultation for Quarter-2, 2020 (Figure 1). Telephone-telehealth dominated usage, especially for shorter consultations (⩽15–30 min) with greater video-telehealth usage in longer consultations (⩾30–75 min) (Figure 2).

## COVID-19-psychiatrist-MBS-telehealth item usage

### New patient assessment psychiatrist telehealth items

For new patient assessment MBS-telehealth items, there were relatively low volumes of usage, representing less than 23%–39% of the combined total of telehealth and face-to-face consultations for Quarter-2, 2020 (Table 1 and Figures 1 and 2).

1. New patient assessments for autism spectrum disorders (item 289 equivalents) were 23% of the Quarter-2, 2019 face-to-face consultations, with video-telehealth used in 65% of these consultations.
2. New patient assessment and 12-month treatment plans (item 291 equivalents) were 27% of the Quarter-2, 2019 face-to-face consultations, with video-telehealth used in 45% of these consultations.
3. Follow-up assessment of 12-month treatment plans (item 293 equivalents) were 40% of the Quarter-2, 2019 face-to-face consultations, with video-telehealth used in 29% of these consultations.
4. New patient assessment items (item 296 equivalents) were 21% of the Quarter-2, 2019 face-to-face consultations, with video-telehealth used in 72% of these consultations.

Face-to-face consultations were generally preferred at 50-72% of the Quarter-2, 2019 level, and when telehealth was used, video-telehealth was preferred, likely due to the need for non-verbal interpersonal cues to better establish empathy and rapport for new patients.^6^

### Standard office-based consultation psychiatrist telehealth items

For MBS-telehealth equivalent to standard time-based office consultations, the majority of the overall increase in consultations were derived from item 300 equivalents, that is, consultations <15 min, representing an 84% increase above the total of Quarter-2, 2019 face-to-face consultations. Of the 300-equivalent telehealth consultations, >87% were delivered by telephone (Table 1 and Figures 1 and 2).

1. Item 302 equivalents, for 15–30 min, were ⩾87% of the face-to-face consultations for Quarter-2, 2019; 79% of 302-equivalent telehealth was by telephone.
2. Telehealth item 304 equivalents represented a significant proportion, 63% of face-to-face consultations for for Quarter-2, 2019, with video used in 31% of consultations.
3. Telehealth 306-equivalent item consultations were 56% of the face-to-face consultations for Quarter-2, 2019, and use of video was 57% of all telehealth.
4. 308-equivalent-item telehealth consultations were 43% of the face-to-face consultations for Quarter-2, 2019, with video used in 53% of telelehealth consultations.
5. 348,350,352-equivalent-item telehealth consultations (*interview of a person other than a patient*) were used for a range of 18%–30% compared to face-to-face consultations for Quarter-2, 2019, with video used in 32%–50% of telehealth consultations.

Shorter duration telehealth consultations (item 300–302 equivalents: ⩽15–30 min) comprised the bulk of telehealth usage, while less telehealth was used for longer consultations. Shorter consultations are used to provide urgent care for patients, as well as addressing patient enquiries about treatment (e.g. medication review), and are thus likely to unprecedently and additionally quantify telehealth, especially telephone, consultations not previously reimbursed by the MBS,^6^ yet may be limited by lack of non-verbal cues affecting the length of consultation.^6^ Video-telehealth provides more non-verbal cues for empathy and rapport, potentially explaining the increased video-telehealth usage for longer consultations (item 304–308 equivalents: 30–75 min), for assessment, management and psychological therapy.

### Group psychotherapy psychiatrist telehealth items

Group psychotherapy consultations were little used, likely due to the combination of their phased introduction through April and that face-to-face consultation remains preferred for therapy (Table 1 and Figures 1 and 2).

## Limitations

COVID-19-psychiatrist-telehealth usage needs to be interpreted with caution due to variations in private practice across Australia. The phased introduction of COVID-19-psychiatrist-telehealth items with varying restrictions to bulk billing until 20 April 2020 is likely to have negatively impacted on usage by private psychiatrists due to income reduction, and thus encouraged maintenance of face-to-face consultations.

## Practical considerations affecting COVID-19-psychiatrist-MBS-telehealth item usage

Practical considerations affecting the uptake of the new COVID-19 MBS-telehealth items include: understanding of the usage of the items; technology, accessibility and cybersecurity; patient and psychiatrist consultation preferences; appropriateness of telehealth for individual patients’ circumstances and suitability to develop empathy and rapport.^6^

## Implications for future private psychiatric care

Rapid adaptation to the outpatient provision of psychiatrist telehealth has also been demonstrated in the US private psychiatric healthcare system^7^ and is likely to have occurred in Australia. During and post-COVID-19, the flexibility and usefulness of psychiatrist telehealth may include: limiting direct patient contact in emergency departments, as well as increasing opportunities for care of isolated patients with serious mental illness and opportunities of private psychiatrists to work with GPs and other mental health practitioners (nurses, social workers, occupational therapists) in telehealth-enhanced care.^8^ Advantages for patients include effectiveness, accessibility and convenience of telehealth, and reduced costs and time for attending consultations in-person.^9^ Further adaptation of psychiatrist telehealth will require significant systemic change, as well as a awareness that cultural, health and socioeconomic factors need to be carefully considered to avoid disparities in care.^8^

## Conclusion

There has been a rapid, flexible and significant uptake of new COVID-19-psychiatrist-MBS-telehealth items, during Quarter-2, 2020 of the pandemic, representing an overall 14% increase in the level of service provided compared to previous office-based consultations for Quarter-2, 2019. Telephone-telehealth usage is predominant for shorter consultations (⩽15–30 min). Provision of in-depth care during assessment, *interview of a person other than a patient* and longer consultations (⩾30–75 min) involved more video-telehealth. Face-to-face consultations were notably lower in Quarter-2, 2020 compared to Quarter-2, 2019, with telehealth comprising over 51% of consultations towards the total.

While telehealth has facilitated commensurate levels of psychiatrist consultations for patients compared to the same quarter in 2019, it also provided scope for expansion of service, despite the phased, bulk-billing–constrained introduction of MBS items. The explanations for the degree of the increase in services might include increased demand due to COVID-19–related distress, quantification of previously non-reimbursed telehealth consultations, as well as the limited expansion of services by private psychiatrists already close to capacity before the pandemic.

Longitudinal analysis of COVID-19-psychiatrist-MBS-telehealth item usage through the pandemic will help identify further factors impacting on usage, including outbreaks of infection (e.g. the second wave in Victoria, July–October 2020) limiting face-to-face consultation. Analyses of MBS-telehealth service outcomes, satisfaction with services, and patient and psychiatrist preferences are needed to assess the quality of care.

In the context of the pandemic in Australia, with ongoing outbreaks, social-distancing and border restrictions, continuing COVID-19-psychiatrist-MBS-telehealth item usage is, based on our findings, essential and likely will continue to be useful in the future.
